# INS/GNSS Integration for Aerobatic Flight Applications and Aircraft Motion Surveying

**DOI:** 10.3390/s17050941

**Published:** 2017-04-26

**Authors:** Edgar L. v. Hinüber, Christian Reimer, Tim Schneider, Michael Stock

**Affiliations:** 1 iMAR Navigation GmbH, D-66386 St. Ingbert, Germany; 2 Stock Flight Systems, D-82335 Berg, Germany; Michael@stockflightsystems.com

**Keywords:** inertial navigation, INS/GNSS, data fusion, aerobatic flight, gravimetry, MEMS gyro, Red Bull Air Race, FOG, RLG, HRG

## Abstract

This paper presents field tests of challenging flight applications obtained with a new family of lightweight low-power INS/GNSS (*inertial navigation system/global satellite navigation system*) solutions based on MEMS (*micro-electro-mechanical- sensor*) machined sensors, being used for UAV (*unmanned aerial vehicle*) navigation and control as well as for aircraft motion dynamics analysis and trajectory surveying. One key is a 42+ state extended Kalman-filter-based powerful data fusion, which also allows the estimation and correction of parameters that are typically affected by sensor aging, especially when applying MEMS-based inertial sensors, and which is not yet deeply considered in the literature. The paper presents the general system architecture, which allows iMAR Navigation the integration of all classes of inertial sensors and GNSS (*global navigation satellite system*) receivers from very-low-cost MEMS and high performance MEMS over FOG (*fiber optical gyro*) and RLG (*ring laser gyro*) up to HRG (*hemispherical resonator gyro*) technology, and presents detailed flight test results obtained under extreme flight conditions. As a real-world example, the aerobatic maneuvers of the World Champion 2016 (Red Bull Air Race) are presented. Short consideration is also given to surveying applications, where the ultimate performance of the same data fusion, but applied on gravimetric surveying, is discussed.

## 1. Introduction

iMAR Navigation GmbH, located in the southwestern part of Germany, is known as a designer and manufacturer of precise and reliable inertial measurement systems for navigation, surveying, tracking and control for more than 20 years, now. iMAR’s systems are used in industrial, automotive, airborne, mapping, marine, defense and research applications worldwide. Due to their extensive experience with all usual inertial sensor technologies, data fusion algorithms and system integration, iMAR is a leading provider of standard and customer-specific inertial measurement and tracking solutions.

**Figure 1:**
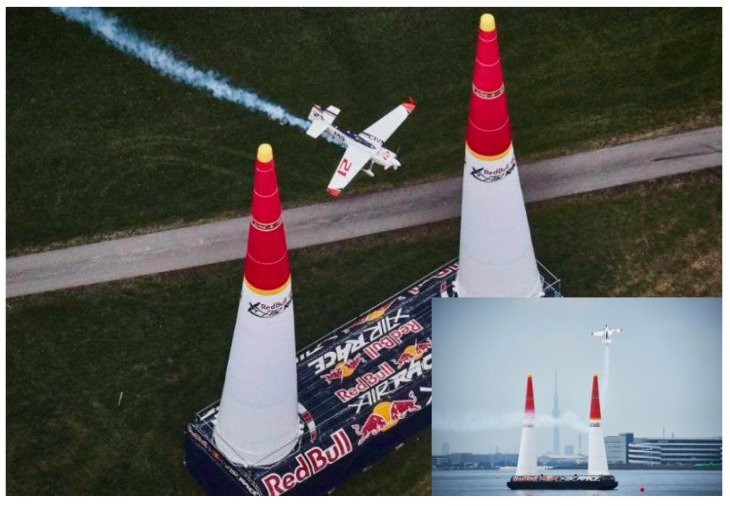
Red Bull Air Race.

This paper presents the technology behind the new generation of iMAR’s iNAT systems. As an example for this paper, flights performed during the Red Bull Air Race 2016 (Figure 1) in both Ascot, UK and at Lausitzring, Germany in the Master Class [1] are shown, and are also compared with the results for a well-known competing system from the market. For this purpose, the iNAT-M200/SLN was mounted on the aerobatic aircraft of Matthias Dolderer, the German participant in the Master’s League and the World Champion 2016 of the RedBull AirRace [2]. These flights are very interesting because they show the most challenging demands of aerobatic flight conditions for an inertial navigation system, demonstrating how the described INS/GNSS solution succeeds, and why the competing system fails dramatically in this environment. The motivation of this article is not to describe the technical insights of the data fusion, but to support the reader, who might intend to use an inertial measurement system, e.g., in his own challenging scientific applications, to be able to understand the requirements of sensor performance, signal processing and sensor data fusion better.

The same system architecture, but equipped with much higher-performance inertial sensors (ring laser gyro technology, RLG) is described as a brief overview, together with flight results, in the last chapter of this paper to show the high accuracy of the developed and implemented hard- and software.

The paper describes in general terms the hardware architecture behind of such modern INS/GNSS implementation, as well as the software architecture. The foundation of strapdown INS/GNSS signal processing is, e.g., given in [3,4], but the challenge is to achieve robustness, performance and simplicity in usage within a small and comparatively low-cost system setup that is easy to operate. To achieve reliable results even during GNSS outages, some approaches, like [5], use additional information like map-based altitude measurements, while our goal is to achieve the best possible results without any additional aiding information, but with a more sophisticated GNSS outlier detection with a tightly coupled data fusion, which also considers the trajectory dependent states observability.

Other publications [6] focus on well-known data fusion with integrity monitoring, using low cost sensors and low power consuming computation, but they do not consider so far aging effects of such low cost sensors in depth, which is nevertheless of high importance especially using commercial grade MEMS based inertial sensors.

Nevertheless our implementation also contains several states inside the extended Kalman filter to be able to use magnetic heading and true airspeed as additional aiding source, we believe that the usage of these data makes it quite complicated for the user to setup a “plug & play” implementation on an UAV or surveying aircraft. Especially on small vehicles, the hard iron and soft iron impacts due to even small changes of the payload during the mission may have a strong impact on magnetic data accuracy while a recalibration of the magnetic effects may not be possible during the mission [7] and therefore for the surveying applications as discussed in this paper the pure INS/GNSS approach is probably a better choice for such applications.

Many other approaches have been published that aim to achieve a higher performance in inertial measurement systems by combination of multiple sensors, partially even with redundant or complementary sensor technologies [8]. The drawback might be a reduced mean-time-between- failures (MTBF), due to a larger number of implemented sensors and the space required for implementation. Therefore the approach given in the present paper followed also the goal to keep the implementation as easy and robust as possible, nevertheless using advanced algorithms that are also able to process very high dynamic motions, like advanced coning compensation [9].

Finally, Chapter 6 of this paper deals with the same INS/GNSS approach as described in the previous chapters, but using a high-performance ring laser gyro-based INS/GNSS setup for gravimetry surveying. Such accurate gravimetry measurements are, e.g., required to aid inertial navigation systems [10], but the focus of Chapter 6 is placed not on INS (*inertial navigation system*) assistance but on the data acquisition of accurate gravimetry data, e.g., to generate a gravimetry model for later assistance. Details regarding data acquisition and data analysis using the iMAR hardware platform are given in [11].

## 2. System Architecture—Hardware

All systems discussed here feature a similar hardware architecture, which differs only in the performance of the inertial sensors used, the data-acquisition setup and the variety of data output interfaces. This means, that the embedded computing, calibration, signal processing and output protocol modules stay the same throughout the entire product lineup.

**Figure 2:**
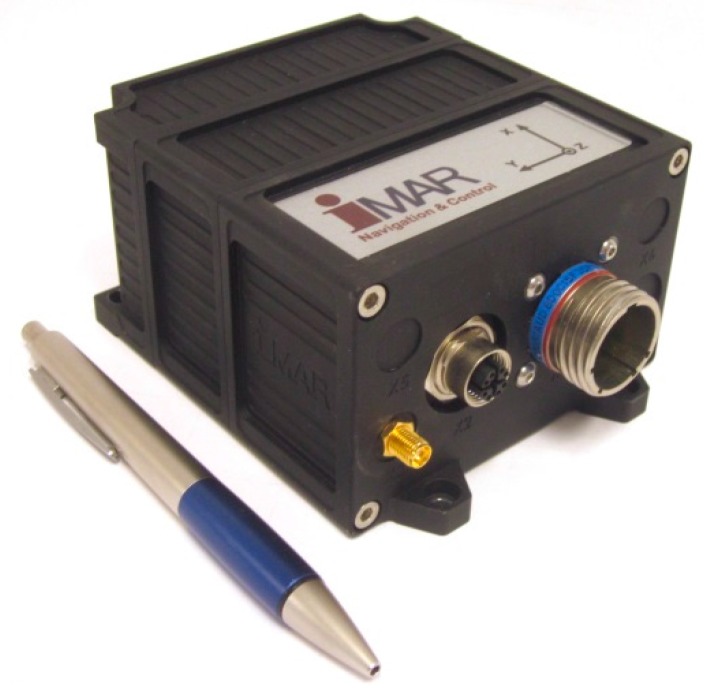
iNAT-M200/SLN (INS/GNSS based inertial navigation, surveying and control system).

The iNAT-M200 INS/GNSS system, which is used to acquire the measurement results being discussed in the following chapters, is designed for navigation, localization and attitude/heading measurement as well as for performing control tasks of manned and unmanned vehicles like UAVs (*unmanned air vehicles*), AUVs (*autonomous underwater vehicles*), UGVs (unmanned ground vehicles) or UMVs (*unmanned marine vehicles*). With its light weight and low power consumption (0.75 kg and 8 W) the iNAT-M200/SLN (Manufacturer iMAR Navigation GmbH, St. Ingbert, Germany) (see Figure 2) is also suitable for small unmanned vehicles. Nevertheless, it is equipped with an integrated high-grade MEMS-based inertial sensor assembly, an integrated GNSS receiver (up to L1L2 GPS + GLONASS + GALILEO & RTK), an integrated wheel sensor (odometer) interface (A/B RS422 level), a powerful integrated miniaturized strapdown computer with data fusion (42+ state Kalman filtering) and all commonly-desired interfaces (Ethernet, UART RS422/RS232, CAN, USB, SYNC) to communicate with external systems. The iNAT-M200 supports all usual data protocols like TCP/IP, UDP, NMEA183, CANaero, ARINC825 and many others, as well as customized communication layers. Additionally, ARINC429 is available on the systems of type iNAT-RQT, iNAT-RQH, iNAT-FSSG, iNAT-FSLG and iNAT-HQS, which cover all inertial sensor technologies from MEMS over fiber optic gyros (FOG), hemispherical gyros (HRG), to the highest-performance ring laser gyros (RLG).

**Figure 3:**
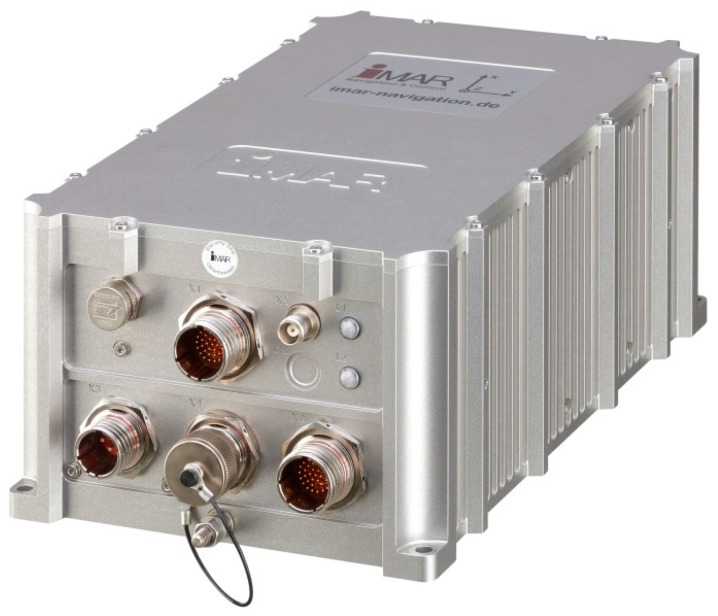
iNAT-RQH type INS/GNSS, based on ring laser technology.

The next picture (Figure 3) shows the iNAT-RQH-4001 (same manufacturer as before), a compact high performance class INS/GNSS system with ring laser gyroscopes and gyro compassing capability as it is used, for example, by Airbus Helicopters (Eurocopter) as master flight reference system since 2016. It is used, for example, to survey the aircraft’s trajectory when performing flight tests and aircraft approvals, but is also used e.g., for subsea applications (with optionally integrated atomic clock to provide most accurate timestamps on all data even during GNSS outages lasting several days, weeks or months).

**Figure 4:**
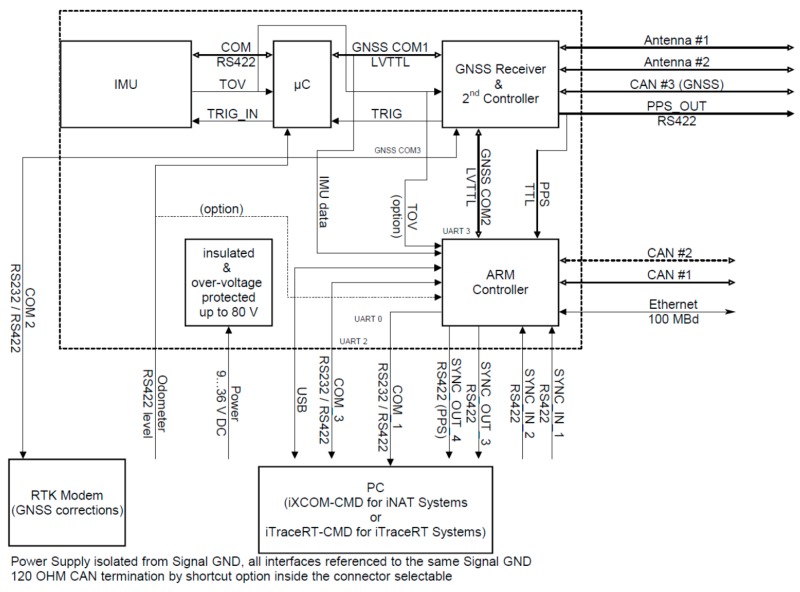
iNAT-M200 Block Diagram.

Figure 4 shows the block diagram of the iNAT-M200 system. The high system accuracy achieved is a result of the high performance of the inertial sensors used, the good performance of the used GNSS engine, the highly sophisticated strapdown and INS/GNSS data fusion algorithms [3,4,9], and the excellent time synchronization inside the mixed FPGA/µC architecture. The time synchronization between the inertial data, in particular, which are sampled with up to 1 kHz, and the GNSS data, which are provided by a common GNSS engine with typical latencies of up to 500 ms, is most important. This is guaranteed by time stamping all measured data with better than 1 µs and by proper processing in a timely fashion. The relevance is illustrated by the following example: At a speed of, e.g., 100 m/s an uncompensated latency of only 1 ms would already lead to a position deviation of 0.1 m, which is a lot if we consider the use of RTK (GNSS correction) in surveying applications to achieve 2 cm position accuracy on the GNSS engine by itself. 

Therefore, in order to provide a 2 cm accuracy in real-time without the lack of GNSS data latency, each system contains a powerful pipeline architecture, which is independent from the inertial sensors, as well as from the GNSS engine.

The main technical data of the both systems iNAT-M200/SLN and iNAT-RQH, which are used in the described applications are given in Table 1.

Aside from high performance, low weight is always a strong requirement for aviation components [7]; the iNAT-M200/SLN is also available as an “OEM” component (without enclosure), where the weight is less than 350 g.

Detailed information about the iNAT systems can be found on www.imar-navigation.de.

## 3. System Architecture—Software

All iNAT systems feature a common firmware which differs only in the sensor-readout module while the calibration, signal processing and output protocol modules stay the same throughout the entire iNAT system family. This ensures complete behavioral compatibility between different device classes, allowing customers to easily switch between INS performance classes from 0.002 deg/h gyro drift up to 1 deg/s gyro drift without even touching their interfacing software.

**Figure 5:**
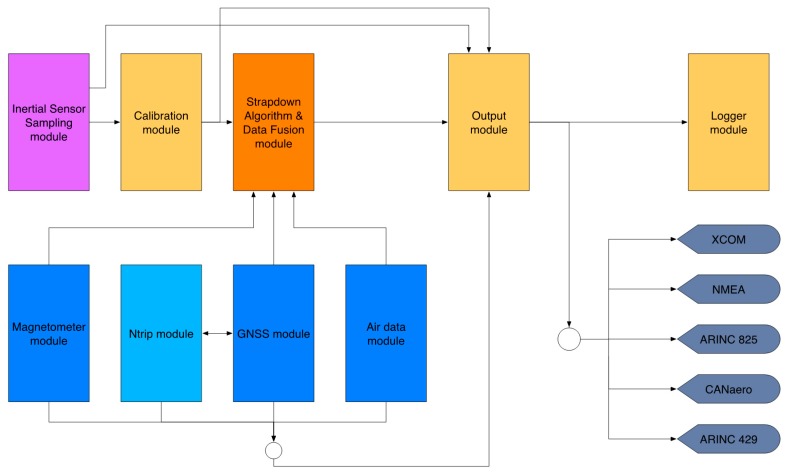
iNAT software module architecture (excerpt).

The software module architecture is depicted in Figure 5, where the software itself is operated on a real-time operating system. After sampling the inertial sensors, sensor and system-level compensation algorithms are performed on their outputs (the so-called calibration module, where sensor misalignment, non-linearity and bias are compensated for over temperature). The compensated data are fed to the coning and sculling compensation to reduce the data rate without significant loss of information [9], and then used to drive the strapdown and extended Kalman filter algorithms [3,10] to produce an optimal INS solution. The data fusion also contains a powerful outlier detection, which is employable on all available assisting sensor sources and which minimizes the usage of unacceptable distorted assisting information within the extended Kalman filter. The output module produces outputs for different protocols as described above. The output rate of messages in either format is adjustable to integer divisors of the inertial data rate.

**Figure 6:**
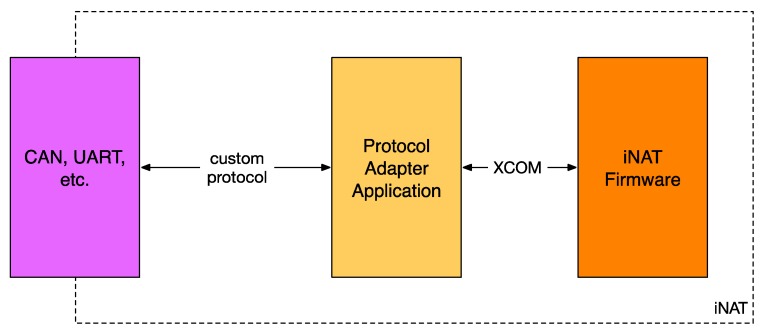
Realization of custom protocols on iNAT systems.

The modular software architecture enables iMAR to develop customer-specific applications running on the IMS (*inertial measurement system*, here used as a generalization of INS, which means *inertial navigation system*) itself which may be used to convert from a customer-specific protocol to iXCOM and back (see Figure 6), easing the development of form, fit and function replacement of legacy hardware with an iNAT IMS.

Another integral part of the iNAT series is the embedded data recorder functionality. All iNAT systems employ a non-volatile memory-based storage capacity of 32 GB. This data recorder may be set up to log timestamped raw inertial data at the full IMS data rate, as well as raw GNSS data for post-processing, as well as all other logs available in the iXCOM protocol, like magnetometer data, airdata, odometer data, DVL (*Doppler velocity log*) data etc. Furthermore, logging of CAN traffic and GNSS correction data received via the integrated NTRIP client may be enabled. All logged data can be retrieved from the system through an FTP connection for easiest use.

**Figure 7:**
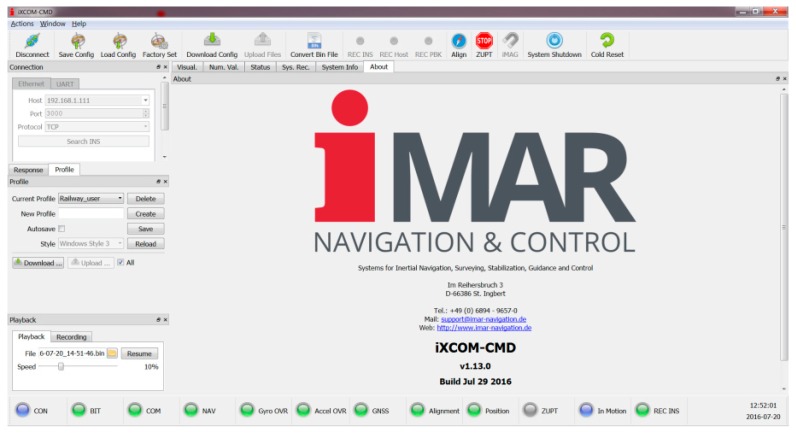
iXCOM-CMD Graphical User Interface (GUI).

For system configuration and data visualization, iMAR provides the iXCOM-CMD GUI software for MS Windows^TM^, Linux and MacOS (Figure 7).

**Figure 8:**
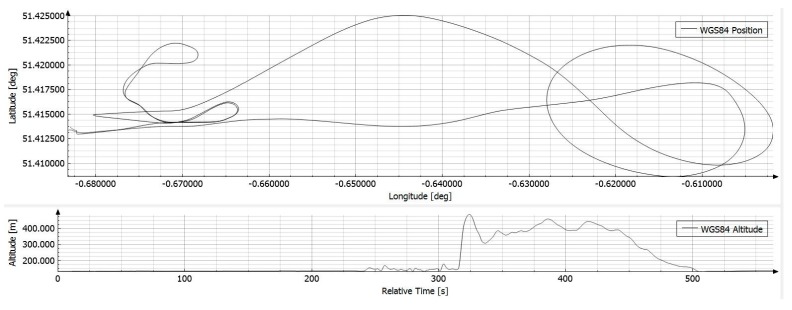
iXCOM-CMD generated plot of flight trajectory.

**Figure 9:**
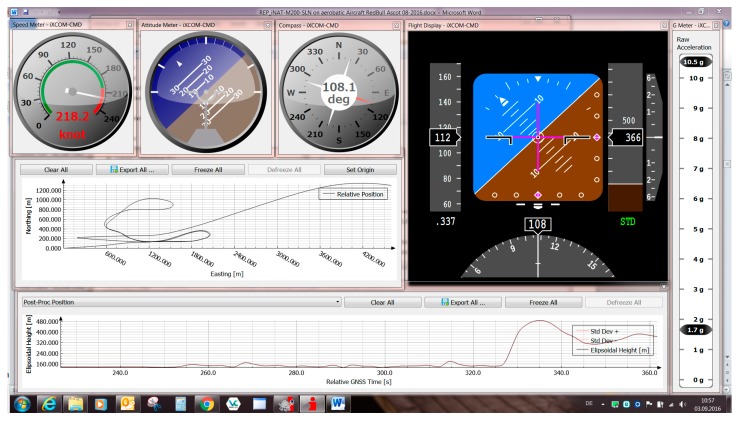
Graphical Instruments and Plots inside iXCOM-CMD.

**Figure 10:**
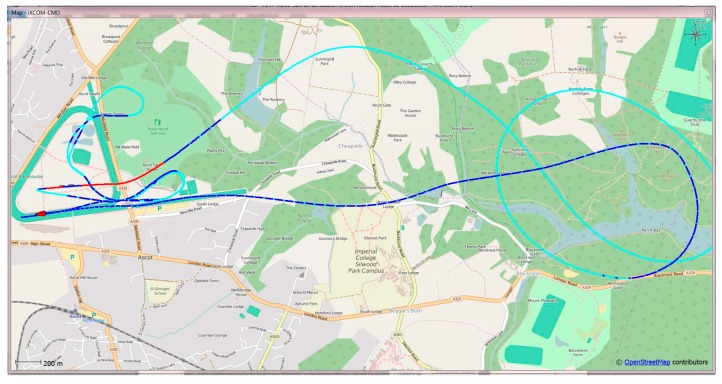
Moving Map of iXCOM-CMD incl. INS/GNSS status visualization.

This software communicates with the iNAT device over the iXCOM protocol, and may also be used to download data from the integrated non-volatile memory by the integrated FTP client. A great number of visualization options is available (Figure 8, Figure 9 and Figure 10), including an artificial horizon, g-meters and speed gauges. The horizontal position can be visualized on a powerful OpenStreetMap-based moving graphical map with both online and offline map support capability. Figure 10 shows a screenshot of the qualification race of Red Bull Air Race in August 2016 in Ascot/UK, obtained with an iNAT-M200 system; Figure 8 shows the trajectory as horizontal and as altitude plot, also within iXCOM-CMD.

## 4. System Architecture—Signal Processing

After acquisition of the inertial data and compensating for mechanical misalignment and temperature-dependent errors, they are processed using the strapdown algorithm to propagate the INS solution. The strapdown algorithm uses an ECEF (*earth-centered-earth-fixed*) mechanization with a quaternion attitude representation, allowing polar operation and attitude propagation even under aerobatic maneuvering.

The output of the strapdown algorithm is then converted to the familiar curvilinear position and Eulerian angles. The inertial drift errors of the strapdown algorithm due to sensor errors are continuously corrected by the output of the extended Kalman filter module, which integrates measurements from different aiding sources, such as GNSS, magnetometer, air data, or odometer, to achieve an optimal solution.

While state-of-the-art implementations for low-weight UAV INS/GNSS solutions [7] still work with, for example, only 16-state Kalman filters, the 42+ state extended Kalman filter used here is based on tightly-coupled data fusion and comprehensive outlier detection and isolation. A significant performance improvement is also achieved due to the estimation of typical aging and hysteresis affected parameters of MEMS inertial sensors like scale factors or misalignments.

**Figure 11:**
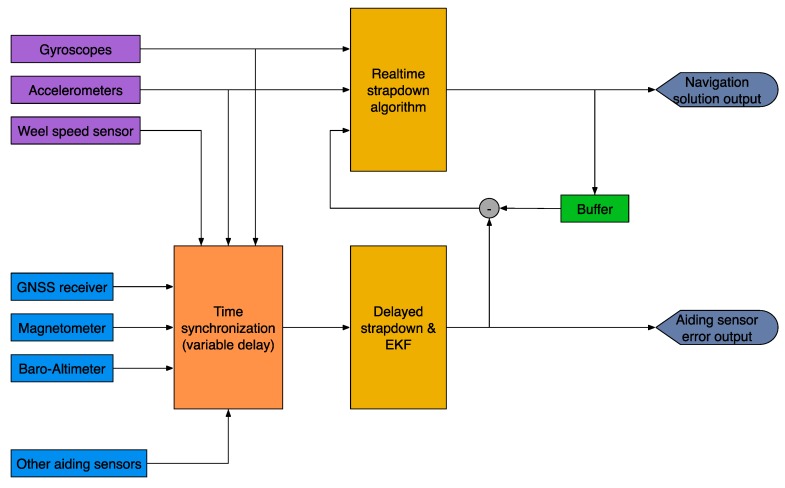
iNAT Signal Processing—Data Fusion.

Figure 11 depicts the data flow between the realtime-strapdown algorithm and the Kalman filter module. The Kalman filter operates on buffered data because the availability of measurements is usually delayed with respect to their time of validity (e.g., up to 0.4 s between the PPS (*pulse per second*) hardware signal of the GNSS receiver and the GNSS solution output via UART for some receivers). All measurements are timestamped with better than 1 µs accuracy and are assimilated in the EKF at their exact time of validity (TOV).

Depending on availability, in aviation applications, Kalman filter-based data fusion makes use of measurements from the integrated GNSS engine, barometric altitude and airspeed measurements from a connected air data computer and magnetic field measurements. Because of the tightly-coupled operation of the Kalman filter, assistance with GNSS is possible even when less than four GNSS satellites are available. The Kalman filter also includes states to compensate for barometric offsets with respect to the GNSS-referenced height above ellipsoid. Calibration of the magnetometer’s soft and hard iron errors, as well as misalignment between the IMS and the magnetometer, is accomplished by an easy-to-use integrated calibration tool, but is not used in the described application to keep the usage as easy as possible.

The parametrization of the extended Kalman filter is specific to the used IMS performance, i.e., for each class (family) of inertial sensors an individual parameter set is worked out. This is based on a significant volume of measurements acquired on our turntables at the iMAR lab using many angular rates, orientations and temperatures; measurements at our hexapod (6 DOF motion platform); and real-world vehicle tests in urban canyons, on the highway, on the proving ground or on our UAV. Additionally, AllanVariance-based data analysis is used for adjusting reasonable margins within the data fusion to achieve the best compromise between system robustness and system accuracy for the desired application.

Each inertial measurement system has to be aligned to the coordinate system in which it shall provide measurements. Typically, such an alignment is performed as a ground alignment at standstill, e.g., on the taxiway of the airport. The iNAT (*navigation and timing*) systems, however, additionally employ a sophisticated “In-Motion Alignment” routine, which allows the system to stay even in-motion during the alignment. This algorithm does not impose any constraints on the type of motion during the alignment, thereby considerably decreasing the burden on the aircraft operator to keep track of the IMS state during initial ground operations and even allowing an in-flight realignment during dynamic maneuvering. This is achieved by transforming the estimated vectors of earth rate and gravity to a locally-stabilized coordinate system, hence reducing the motion dynamics influences. It has been shown that the algorithm can be used both in static and dynamic environments without loss of performance, thus making the handling of alignment as easy as possible for the operator.

## 5. Results during Red Bull Air Race

iMAR has been partnering with the aerobatic pilot Matthias Dolderer (Germany) and Stock Flight Systems for trajectory optimization in the Red Bull Air Race [1] since its reconception in 2014.

In the Red Bull Air Race, the pilots have to navigate a challenging obstacle course in the fastest time. They fly individually against the clock and have to complete tight turns through a slalom course consisting of pylons, known as “Air Gates”. During this course, turn rates reaching up to 400°/s and load factors of up to 10 g act on the plane, the pilots, and on the INS/GNSS system used.

Matthias Dolderer’s team MD21 [2] uses the CANaerospace Data Acquisition and Recording System (CDARS) from Stock Flight Systems, with the inertial navigation solution provided by an iMAR iNAT-M200/SLN. Together with numerous other pieces of measurement equipment, the CDARS installation in MD21’s Zivko Edge 540 V3 RSR race plane provides unprecedented potential for optimization of flight trajectory, flight dynamics and coordination of pilot controls and engine management. All measurement and control units interact with each other using two 1 MBit/s CANaerospace networks with a miniaturized and lightweight data logger recording all data synchronously.

**Figure 12:**
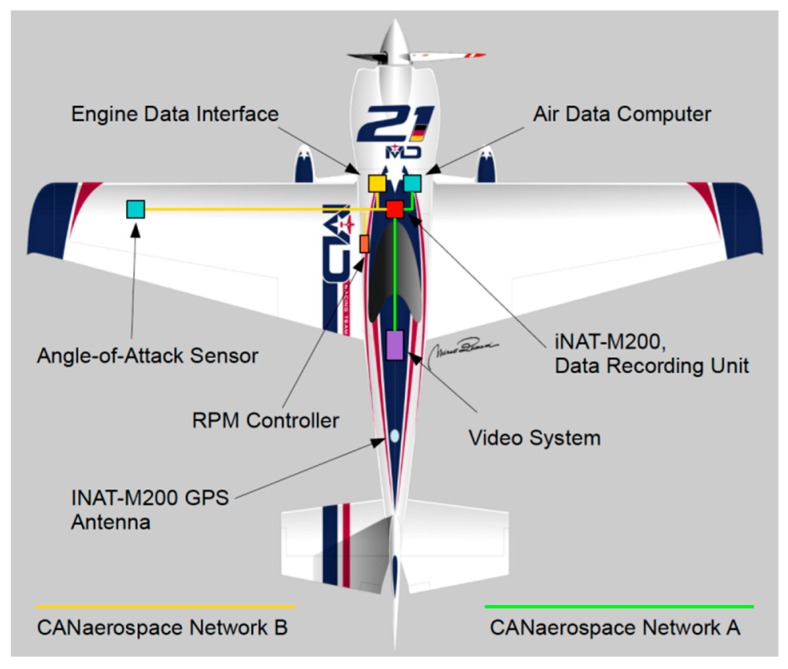
Setup of measurement systems on the race plane.

Figure 12 shows a block diagram of the most important measurement systems on the airplane. The logged data can be extracted and analyzed with the highly specialized Air Race Tool (ART), which displays pylon positions, the flown trajectory and, for every point on the trajectory, the current kinematic state of the airplane (3D velocity and attitude, acceleration, and angular rate, obtained from the iNAT-M200), as well as important flight parameters like true airspeed or engine RPM. This complete assessment of aircraft state enables MD21’s race engineers and tacticians to identify crucial spots in the track, and thus enables split seconds to be shaved from the time even in the very short breaks in between subsequent runs of training, qualifying and the race.

**Figure 13:**
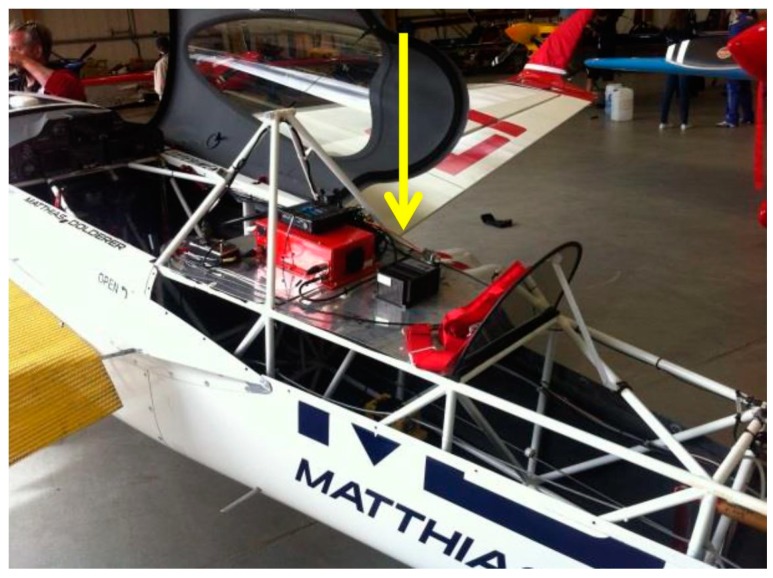
iNAT-M200 in the race plane.

Figure 13 shows the integration of the iNAT-M200/SLN into the race aircraft at Tannheim Airport. 

**Figure 14:**
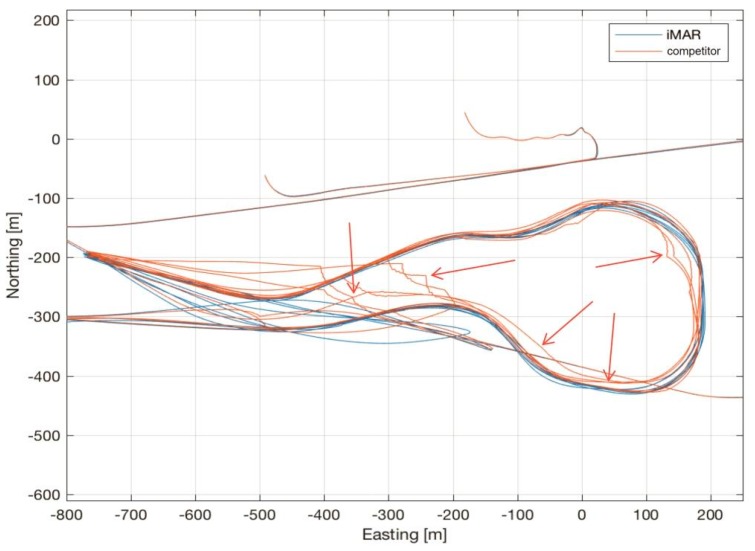
Comparison of trajectory—iMAR navigation vs. competitor’s solution on the same aircraft (Lausitz).

Figure 14 shows the trajectory recorded by the iMAR iNAT-M200/SLN during free training for the Air Race at Lausitzring on 3–4 September 2016, together with the trajectory recorded by a competitor’s INS/GNSS, which had been used by RedBull’s race organizer as the official tool to measure the trajectory (both systems had always been installed on the same aircraft).

**Figure 15:**
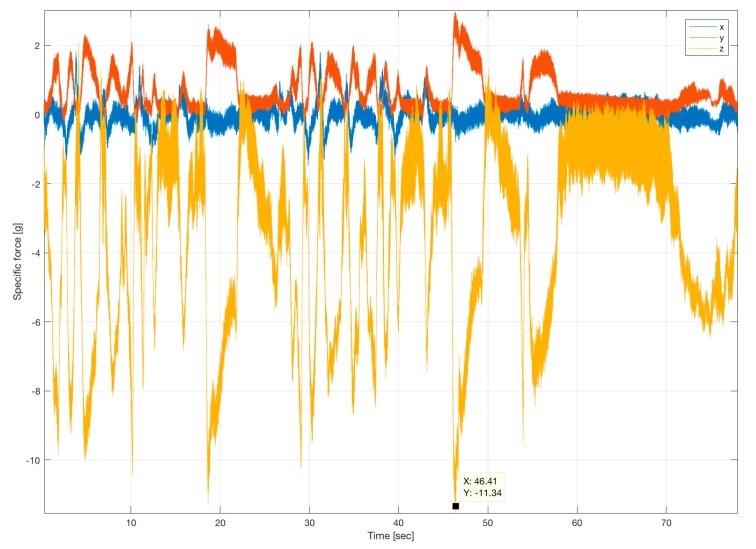
G-Forces [g] experienced over Time [s] during a training run of Red Bull Air Race (*x* = aircraft’s roll axis, *y* = aircraft’s body right axis, *z* = aircraft’s body downward axis).

As shown over a long history of flights, iMAR Navigation’s iNAT-M200 provides superior results, even under these extremely harsh conditions with several high-g turns (peaking at over 11 g, see Figure 15) and prolonged GNSS outages of up to 15 s or more during loops and barrel rolls.

While these conditions do not lead to any discontinuities in iMAR’s navigation result, the competitor’s product showed much bigger drift during these outage periods, and several big correction impulses of their data fusion after the end of these outages (see red arrows in the plot, which show the strong deviations of the competing navigation system), rendering any kind of detailed trajectory analysis impossible. Due to that, team MD21 relied entirely on the iMAR system for the Red Bull Air Race season 2016, which eventually led to Matthias Dolderer taking the World Champion title one race before the end of the season.

**Figure 16:**
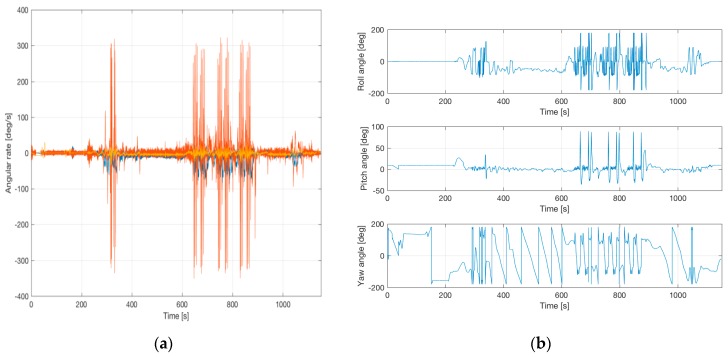
Roll, Pitch and Yaw and angular rates during aerobatic flight. (**a**) shows the very high roll rates, (**b**) shows e.g., the periods of “head-down” and “nose up”.

Figure 16 shows attitude and heading during such flight. A roll angle of 180 deg or −180 deg indicates a “head down” flight, where also the GNSS antenna was oriented to the ground (and hence no GNSS satellites are in view). The angular rates show the high dynamics, especially on the roll axis, which also leads to very high centripetal acceleration impacts, which must be well handled inside the strapdown computation to avoid related deviations.

**Figure 17:**
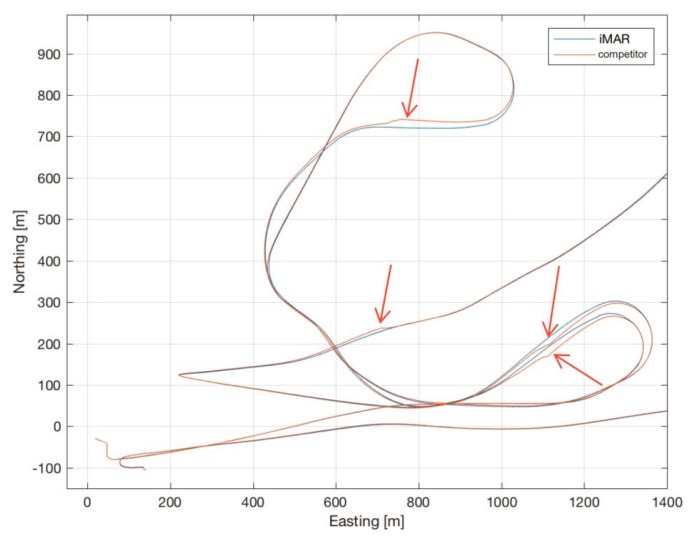
Comparison of trajectory—iMAR navigation vs. competitor’s solution on the same aircraft (Ascot).

Figure 17 shows similar results from the ASCOT race in August 2016. Here we see deviations (see the parts of the trajectory of the competitor’s trajectory, where strong deviations are obvious, marked with red arrows) of up to 50 m and more for the competing product.

Another important advantage of iMAR’s implementation over the competing product is that, due to iMAR’s novel In-Motion alignment method, a dual antenna heading solution is not required (and hence not used) for robust heading initialization and the pilot is not required to wait for the alignment of the INS to complete before taxiing or takeoff.

**Figure 18:**
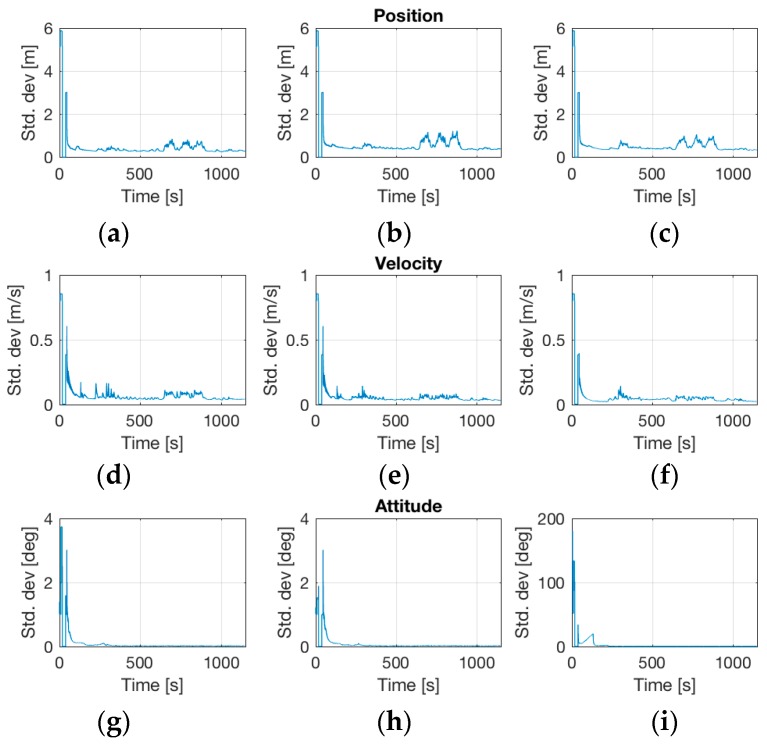
Estimated deviations of navigation solution. (**a**–**c**) Standard Deviations of Position in East/North/Down; (**d**–**f**) Standard Deviations of Velocity in East/North/Down; (**g**–**i**) Standard Deviations of Roll, Pitch and Yaw.

Figure 18 shows the estimated accuracy of position (better than 1 m RMS, coordinate system NED), velocity (better than 0.1 m RMS), and attitude (roll, pitch better than 0.05 deg RMS, heading better than 0.1 deg RMS). Take into account that all the presented results of the iNAT-M200 system had been acquired and provided in real-time on the race aircraft, without any post-processing. No air data information or magnetic heading information had been used and only a single GNSS antenna had been applied on the aircraft (this makes the implementation on the aircraft much easier due to minimum cabling). GPS and GLONASS had been taken with L1L2 and standard WAAS/EGNOS corrections via satellite. The performance class of the used inertial sensors had been <0.1 deg/s gyro bias (day-to-day, over temperature range), <0.5 deg/h bias instability at constant temperature (derived from AllanVariance analysis), <15 deg/h bias instability gyro bias instability (derived from AllanVariance analysis over changing temperature), 6 mg accelerometer bias (day-to-day) and <0.1 mg accelerometer bias instability at constant temperature (also derived from AllanVariance analysis).

On request, the system can be upgraded onsite to have RTK capability for providing sub-decimeter level accuracy where needed.

## 6. Airborne Gravimetry with iNAT-RQH

iNAT inertial measurement systems are deployed in a very wide range of applications. One particularly demanding type of application, requiring the highest classes of inertial sensor performance, is airborne gravimetric measurement using the inertial sensors from the INS without needing to use specialized gravimetric equipment. Such data are, for example, required for gravity modeling to be applied for free-navigating INS [4].

**Figure 19:**
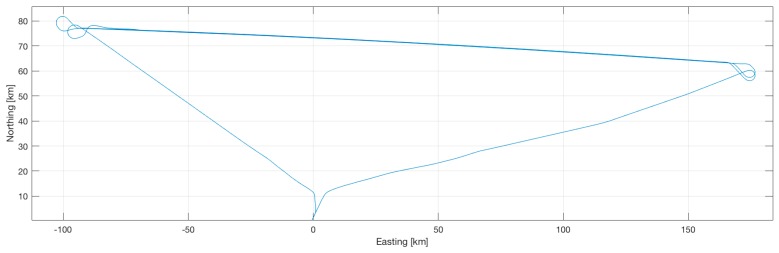
Trajectory profile of an airborne gravimetry test flight.

**Figure 20:**
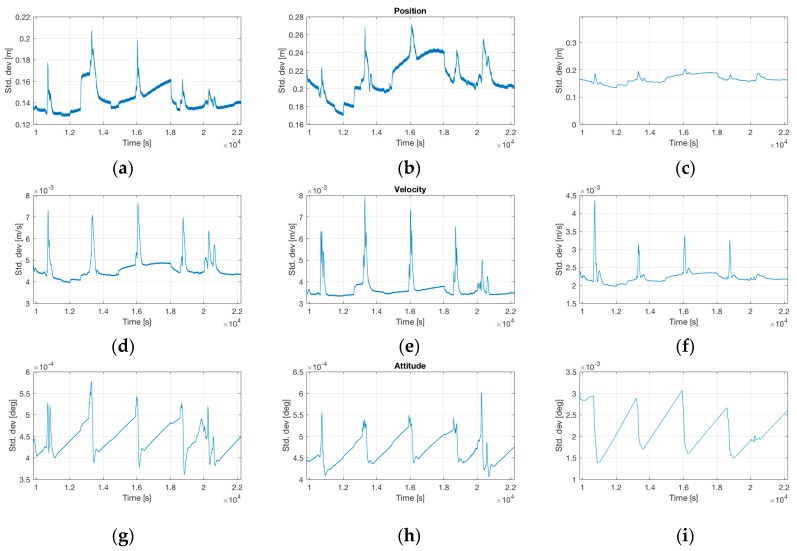
Standard deviations of navigation state during the test flight. (**a**–**c**) Standard Deviations of Position in East/North/Down; (**d**–**f**) Standard Deviations of Velocity in East/North/Down; (**g**–**i**) Standard Deviations of Roll, Pitch and Yaw.

Online results from iMAR’s Kalman-filter-based data fusion of GNSS and inertial data showed static alignment errors of less than 0.03 deg in heading and a navigation performance of significantly better than 0.2 nm/h. Online results are depicted in Figure 19 and Figure 20. The flight had been performed in the USA in early September 2016, the plots of position and velocity are coordinated in NED and attitude as roll/pitch/yaw.

The flight shown in the plots had been performed over about 12,000 s, i.e., 3.3 h. Due to the high performance of the used inertial sensors (ring laser gyroscopes and closed-loop quartz pendulous accelerometers) inside the iNAT-RQH-4001, very low errors in attitude (<0.0006 deg in roll/pitch, <0.003 deg in heading) and velocity (<8 mm/s) are achievable under sufficient dynamic motion with GNSS coverage, even without resorting to post-processing and without RTK assistance (see Figure 20). The performance class of the inertial sensors used was <0.002 deg/h gyro bias (day-to-day, over temperature range), <0.0001 deg/h bias instability at constant temperature (derived from AllanVariance analysis), <25 µg accelerometer bias (day-to-day) and 0.1 µg accelerometer bias instability at constant temperature (also derived from AllanVariance analysis).

These results also make the iNAT-RQH and iNAT-RQT systems very attractive for advanced applications in laser scanning (LIDAR), photogrammetric surveying or SAR (synthetic aperture radar).

Columbia University extensively uses the online-recorded timestamped raw inertial and GNSS data to post-process the data together with external GNSS reference station data as well as non-causal forward-backward processing to obtain gravity measurements reaching an accuracy of better than 2 µg (i.e., 2 mGal), which compares very well with classical gravimetric instruments but for a fraction of the price. A detailed data analysis using such inertial measurement data obtained from iNAT-RQH is described in [11].

## 7. Conclusions

With the iNAT system family, supporting all common areas of INS/GNSS/xxs data fusion, iMAR Navigation has introduced highly precise inertial measurement systems serving applications demanding the complete range of inertial sensor performance. All these systems feature a common hardware and software architecture, making iNAT the system of choice for system integrators in need of different device classes. The implementation of special customer requirements is easy thanks to the modular architecture in both hardware and software. All these systems feature low power consumption and small size while most powerful data fusion for real-time and post-processing solutions is implemented together with advanced inertial sensors.

In this paper, two different kinds of applications had been presented. The paper showed the challenging task of installing a MEMS based INS on an aerobatic airplane. The race plane had to function under extremely dynamic conditions, with up to more than 10 g acceleration and 300 deg/s angular rate. This led to high requirements in terms of inertial sensor bias stability, scale factor accuracy and time stamping in order perform the most accurate signal processing inside the INS in real-time.

To be able to also handle the significant GNSS outages with high accuracy, the results of our innovative and powerful INS/GNSS data fusion containing a 42+ state model were discussed, where the estimation of aging relevant parameters of MEMS-based inertial sensors is a key factor of the success.

The results, obtained with a high-performance INS, using the same data processing, were also demonstrated by experimental results for the application of high performance geodetic surveying in the field of gravimetry.

## Figures and Tables

**Table 1:** Performance Comparison iNAT-M200 and iNAT-RQH.

Parameter	iNAT-M200/SLN	iNAT-RQH-4001
angular random walk	0.15°/sqrt (h)	0.0011°/sqrt (h)
accelerometer noise density	60 µg/sqrt (Hz)	8 µg/sqrt (Hz)
gyroscope day-to-day bias	<0.07°/s	0.002°/h
gyroscope bias instability (AV)	<0.5°/h	<0.001°/h
accelerometer day-to-day bias	<6 mg	<25 µg
accelerometer bias instability (AV)	<0.06 mg	<1 µg
internal data acquisition rate	1000 Hz	1800 Hz
data output rate	500 Hz	300 Hz
sensor range	±400°/s, ±10 g	±500°/s, ±20 g
provided output data	angular rates & accel. (raw data and processed data), GNSS raw data, position & velocity & roll/pitch/yaw/quaternion (from INS/GNSS data fusion), status, sensor temperatures [all data time-stamped regarding to PPS with 1 µs accuracy]	
GNSS receiver (integrated)	L1L2	L1L2
	GPS + GLO + SBAS/EGNOS	GPS + GLO + SBAS/EGNOS
inertial sensor technology	MEMS gyros, MEMS accels.	ring laser gyros, servo accels.
weight	0.8 kg	7.9 kg
